# Reprogramming of *Trypanosoma cruzi* metabolism triggered by parasite interaction with the host cell extracellular matrix

**DOI:** 10.1371/journal.pntd.0007103

**Published:** 2019-02-06

**Authors:** Eliciane C. Mattos, Gisele Canuto, Nubia C. Manchola, Rubens D. M. Magalhães, Thomas W. M. Crozier, Douglas J. Lamont, Marina F. M. Tavares, Walter Colli, Michael A. J. Ferguson, Maria Júlia M. Alves

**Affiliations:** 1 Departamento de Bioquímica, Instituto de Química, Universidade de São Paulo, São Paulo, Brazil; 2 Departamento de Química Analítica, Instituto de Química, Universidade Federal da Bahia, Salvador, BA, Brazil; 3 Departamento de Biologia Celular e Molecular e Bioagentes Patogênicos, Faculdade de Medicina de Ribeirão Preto, Universidade de São Paulo, Ribeirão Preto, Brazil; 4 Wellcome Centre for Anti-Infectives Research, School of Life Science, University of Dundee, Dundee, United Kingdom; 5 Fingerprints Proteomics Facility, School of Life Sciences, University of Dundee, Dundee, United Kingdom; 6 Departamento de Química Fundamental, Instituto de Química, Universidade de São Paulo, São Paulo, Brazil; Liverpool School of Tropical Medicine, UNITED KINGDOM

## Abstract

*Trypanosoma cruzi*, the etiological agent of Chagas’ disease, affects 8 million people predominantly living in socioeconomic underdeveloped areas. *T*. *cruzi* trypomastigotes (Ty), the classical infective stage, interact with the extracellular matrix (ECM), an obligatory step before invasion of almost all mammalian cells in different tissues. Here we have characterized the proteome and phosphoproteome of *T*. *cruzi* trypomastigotes upon interaction with ECM (MTy) and the data are available via ProteomeXchange with identifier PXD010970. Proteins involved with metabolic processes (such as the glycolytic pathway), kinases, flagellum and microtubule related proteins, transport-associated proteins and RNA/DNA binding elements are highly represented in the pool of proteins modified by phosphorylation. Further, important metabolic switches triggered by this interaction with ECM were indicated by decreases in the phosphorylation of hexokinase, phosphofructokinase, fructose-2,6-bisphosphatase, phosphoglucomutase, phosphoglycerate kinase in MTy. Concomitantly, a decrease in the pyruvate and lactate and an increase of glucose and succinate contents were detected by GC-MS. These observations led us to focus on the changes in the glycolytic pathway upon binding of the parasite to the ECM. Inhibition of hexokinase, pyruvate kinase and lactate dehydrogenase activities in MTy were observed and this correlated with the phosphorylation levels of the respective enzymes. Putative kinases involved in protein phosphorylation altered upon parasite incubation with ECM were suggested by *in silico* analysis. Taken together, our results show that in addition to cytoskeletal changes and protease activation, a reprogramming of the trypomastigote metabolism is triggered by the interaction of the parasite with the ECM prior to cell invasion and differentiation into amastigotes, the multiplicative intracellular stage of *T*. *cruzi* in the vertebrate host.

## Author summary

Adhesion of *Trypanosoma cruzi* to distinct elements of ECM involving different surface proteins from the infective stage of the parasite has been described. Despite the relevance of ECM for *T*. *cruzi* infection, the signaling pathways triggered in trypomastigotes upon interactions with ECM are less well understood. In previous work we demonstrated the dephosphorylation of proteins, such as α-tubulin, paraflagellar rod proteins and ERK 1/2 in trypomastigotes incubated with either laminin or fibronectin. Further, we described changes in the S-nitrosylation and nitration pattern of proteins from trypomastigote incubated with ECM. To expand our knowledge on ECM triggered parasite signaling we applied quantitative proteomic and phosphoproteomic studies to trypomastigotes incubated with ECM (MTy) compared to controls (Ty). Our results indicate relevant changes in total protein and phosphoprotein profiles in MTy. The kinases implicated in the modifications were suggested by bioinformatic analyses, as well as the number of modifications and the frequency of amino acids per peptide that have been modified. Proteins involved in metabolic processes, including enzymes from the glycolytic pathway, phosphatases and kinases were the most representative groups among the proteins modified by phosphorylation. Quantification of metabolites in MTy and Ty also indicated that glucose metabolism is impaired in trypomastigotes incubated with ECM. The significant inhibition of hexokinase, pyruvate kinase and lactate dehydrogenase activities in MTy associated with phosphorylation levels, strongly suggests that trypomastigotes reprogram their metabolism in response to interaction with the extracellular matrix, an obligatory step prior to host cell invasion.

## Introduction

The protozoan *T*. *cruzi*, the etiological agent of Chagas’ disease, affects a wide range of mammalian hosts, including humans. It is estimated that 8 million people are infected and 25 million are at risk, most of them living in areas of poor socioeconomic development [1]. The cell cycle of *T*. *cruzi* involves an invertebrate vector (triatomine bugs) and a mammalian host, and well-defined developmental stages (epimastigotes, metacyclic trypomastigotes, amastigotes and blood trypomastigotes). *T*. *cruzi* trypomastigotes, the classical infective stage, invade almost all mammalian cell and tissue types, and invasion is an obligatory step in their life-cycle in mammals. An essential step immediately prior to the mammalian cell invasion is the interaction of the parasite with the surrounding extracellular matrix.

The extracellular matrix (ECM) is a highly dynamic non-cellular three-dimensional macromolecular network, which regulates different cellular functions, such as growth, differentiation or survival [cf. 2]. Its composition includes structural components (“matrisome”, [3]) and elements that can interact with or remodel the ECM [4]. Collagens, proteoglycans and glycoproteins (laminins, fibronectins, thrombospondins, tenascins, among others) constitute the main core of the ECM proteins. ECM-affiliated proteins (such as mucins, syndecans, plexins) and ECM-regulators (such as lysyl oxidases, sulfatases, extracellular kinases, proteases and secreted factors, such as TGFβ, cytokines) were classified as matrisome-associated proteins [4]. Cell-ECM interactions occur mainly by integrins present at the cell surface, which connect the extracellular signals and the intracellular response by the activation of specific signaling pathways [5, 6] and dysregulation of the ECM is associated with the development of several pathological conditions [4,7].

Adhesion of *Trypanosoma cruzi* and other parasites to distinct elements of ECM has been described to involve different surface proteins from the infective stage of the parasite, of which the gp85/transialidase family plays an essential role (rev. [8]). *T*. *cruzi* binds to collagen [9], fibronectin [10–14], laminin [11,15, 16, 17], thrombospondin [11], [18], heparan sulfate [11, 12, 14, 19], galectin-3 [20, 21], as well as TGF-β [22]. Also, remodeling of ECM was observed during infection, with modifications in collagen and fibronectin content, as well as reorganization of laminin [13], at least partially due to *T*. *cruzi* proteolytic enzymes [23–25].

Despite the relevance of ECM for *T*. *cruzi* infection, the signaling pathways triggered by the trypomastigote-ECM interaction are less well known. Recently, we demonstrated a decrease in S-nitrosylation and nitration in the majority of the trypomastigote proteins when parasites are incubated with ECM [26]. In addition, dephosphorylation of proteins, such as α-tubulin, paraflagellar rod proteins (PFR or PAR), as well as ERK 1/2 was observed in trypomastigotes incubated with either laminin or fibronectin [27], although these do not reflect the entirety of possible interactions between parasite and ECM. To have a better understanding of the process, quantitative proteomic and phosphoproteomic approaches were employed to analyze changes in *T*. *cruzi* trypomastigote proteins when the parasites are incubated with ECM. Herein we show important changes in the *T*. *cruzi* trypomastigotes proteome, as well as in protein phosphorylation levels upon interaction with ECM. Proteins involved with metabolic processes, phosphatases, kinases and RNA/DNA binding elements were highly represented among the proteins modified by phosphorylation. In particular, a decrease of the glycolytic pathway was suggested by metabolite quantification and measurement of hexokinase, pyruvate kinase and lactate dehydrogenase activities, suggesting an extensive metabolic adaptation of trypomastigotes prior to host cell invasion. Taken together, our data show that not only structural adaptations but also important metabolic changes occur upon parasite interaction with ECM. Understanding these changes may further elucidate the adaptive mechanisms involved in parasite-host interaction.

## Materials and methods

### *Trypanosoma cruzi* culture and incubation with ECM (Geltrex)

#### Parasite culture

*T*. *cruzi* trypomastigotes, Y strain, were obtained as described [28]. Five days after epithelial cultured cells (LLCMK_2_) infection, trypomastigotes released into the culture medium were collected and spun down at 5,000 *x g* for 10 min. The pellet was resuspended in cold PSG buffer (5 mM NaH_2_PO_4_/Na_2_HPO_4_ pH 8.0, 7.3 mM NaCl, 1% dextrose) and the trypomastigotes were purified by DEAE-cellulose as described [29]. Briefly, DEAE-cellulose column was washed twice with PS buffer (5 mM NaH_2_PO_4_/Na_2_HPO_4_ pH 8.0, 7.3 mM NaCl), followed by three washes with PSG buffer. Parasites were then added to the column, washed with PSG and the purified parasites recovered in the flow through.

#### ECM-treated trypomastigotes (MTy)

Purified trypomastigotes (5 x 10^8^ parasites) were resuspended in 5 mL of Modified Eagle’s Medium (MEM) supplemented with 2% FBS, mixed with 150 μL Geltrex (Geltrex™ LDEV-Free Reduced Growth Factor Basement Membrane Matrix, Invitrogen) in 15 mL tubes, and incubated for 2 h at 37°C and 5% CO_2_. After incubation with ECM, trypomastigotes were spun down (4,000 *x g* for 5 min), the pellet ressuspended in 5 mg/mL collagenase, 10 mM HEPES pH 7.4, 0.36 mM CaCl_2_ and incubated for 15 min at 37°C and 5% CO_2_. After three washes with PBS containing 10 mM EDTA (PBS-EDTA), the parasites were finally washed with PBS-EDTA supplemented with protease and phosphatase inhibitors (0.1 mM NaF, 0.1 mM Na_3_VO_4_, 0.05 mM sodium β-glicerophosphate; SIGMAFAST™ Protease Inhibitor Tablets-SIGMA-ALDRICH; 0.1 mM PMSF), frozen and kept at -80°C.

### Proteome and phosphoproteome strategy

#### Protein extraction and digestion

Frozen pellets of trypomastigotes were resuspended in 200 μL of Lysis Buffer (4% SDS, 25 mM TCEP, 50 mM N-Ethyl Maleimide, 0.01 M NaPO_4_ pH 6.0, 0.1 M NaCl, containing phosphatase and protease inhibitors, as described above). Lysates were sonicated for 15 min (frequency of 30%) and heated to 65°C for 10 min. Lysates were then treated as follows: 100 μL of lysate was treated with 400 μL methanol, 100 μL chloroform and 300 μL milliQ water, with 1 min of strong agitation between the additions. The samples were spun down at 9000 *x g* for 5 min at room temperature, the upper phase carefully removed, 300 μL methanol were added to the pellet and centrifuged as above. The entire supernatant was removed and the pellet was air dried at room temperature. The pellet was resuspended in 200 μL 8 M Urea, 0.1 M Tris-HCl pH 8.0, 1 mM CaCl_2_ and the total protein quantified. The material was digested overnight at 37°C with LysC (Pierce™ Lys-C Protease, MS Grade) at 1:100 ratio (protease: total protein), diluted 8-fold in 1 M Urea, 0.1 M Tris-HCl pH 8.0, 1 mM CaCl_2_, and treated with trypsin (Pierce™ Trypsin Protease, MS Grade) at 1:100 ratio (protease:total protein), for 4 h at 37°C. After the incubation, TFA was added to 1% final concentration and the peptides desalted by Sep-Pak columns (Sep-Pak C18 3 cc Vac Cartridge, WATERS), as manufacturer recommendations. The material was air dried by *speedvac*, resuspended in 50 mM HEPES (pH 8.5) and the peptides quantified by CBQCA (*CBQCA Protein Quantitation Kit*–Molecular probes/Invitrogen) prior to Tandem Mass Tag labeling. Three independent biological samples from parasites incubated or not with ECM were analyzed.

#### TMT-Sixplex incorporation and HILIC fractionation

After protein digestion, peptide labeling was done following the procedures for Tandem Mass Tags labeling (TMTsixplex™ Label Reagent, Thermo Fisher Scientific). TMT-tags (0.8 mg/tag) were diluted in 41 μL of anhydrous acetonitrile (ACN) and mixed for 5 min at room temperature. Each tag was carefully transferred to the peptide samples from trypomastigotes incubated with ECM (MTy = TMT-129, 130, 131) and incubated without ECM (control Ty–TMT-126, 127 and 128).

The peptide-TMT mixtures were incubated for 120 min at room temperature. After incubation, 8 μL of 5% hydroxylamine was added and incubated for 15 min to quench the labelling reaction. Samples were then mixed together in one tube, in a quantity of 270 μg peptide-TMT/sample, a total of 1620 μg. The mixture was then filtered on a Sep-Pak C18, cartridge to remove free tags and then dried in *Speedvac*. The sample was then resuspended in 200 μL 80% ACN, 0.1% TFA. TMT labeled peptides were injected onto a TSKgel Amide-80 column (4.6mm x 25cm, 3um particle size) and guard column (TSKgel Amide-80, 4.6 x 1.0cm, 3um particle size), using a Dionex Ultimate 3000 HPLC system. Buffer A was 0.1% TFA in water and buffer B was 0.1% TFA in 100% acetonitrile. Chromatography was performed at 0.6 mL/min at 30°C, starting at 80% buffer B, reducing to 70% B after 10 min and then to 60%B after a further 30 min and finally to 0% B after a further 5 min (held for 10 min). Buffer B was then increased to 80%B for 35 min for re-equilibration. Fractions were collected every 2 minutes and those with low amounts of peptide were pooled together (Fractions 8 to 11), producing 11 fractions for subsequent LC-MS/MS analysis. An aliquot of 5% of the fraction volume was taken for total proteome analysis while the remaining 95% was taken for phospho-enrichment.

#### TiO_2_-IMAC phospho enrichment

Phosphopeptide enrichment was carried out as described in the TitansphereTmPhos-TiO_2_ Kit manual, using TiO_2_ resin (Titansphere, GL Sciences Inc, Japan) in batch mode. The peptide fractions were resuspended in 80% ACN, 1M Glycolic acid, 5% TFA and then mixed to the resin, previously equilibrated with the same solution. 1 mg of resin was employed for 500 μg of peptide. After the peptide-resin incubation for 20 min at room temperature, the resin was washed three times with 80% ACN, 1% TFA. Finally, the phosphopeptides were eluted with 0.5% NH_4_OH.

#### LC-MS/MS analysis

LC-MS/MS analysis was carried out using an UltiMate 3000 nanoRSLC UHPLC system (Thermo Scientific) coupled to a Q Exactive HF hybrid quadrupole-Orbitrap mass spectrometer (Thermo Scientific) (FingerPrints Proteomic Facility, University of Dundee, Scotland). Seven μL of proteome sample and 15 μL of phosphoproteome sample were injected onto a PepMap nanoViper C18 trap column (100 μm x 2 cm, 5 μm, 100 Å, Thermo Scientific) at a flow rate of 5 μL/min. The trap column was equilibrated in 98% Buffer A (2% ACN, 0.1% Formic acid (FA)) and then after sample injection washed for 10 min with the same flow then moved in line with the C18 EasySpray resolving column (75 μm x 50 cm, PepMap RSLC C18, 2 μm, 100 Å). The peptides were eluted at a constant flow of 300 nL/min with a linear gradient from 5 to 40% Buffer B (80% ACN/ 0.08% FA) over 120 min and column was finally washed with Buffer B for 15 min and 98% Buffer A for 24 min. An ionspray voltage of 1.95kV and a capillary temperature of 250°C was applied to the EasySpray Column via the EasySpray source. The total ms runtime was set at 156min. The Full MS parameters were as follows: MS Resolution at 120,000; MS AGC target value at 3e6; maximum Ion Time at 50 msec and the mass range at 355-1800m/z. The data dependant MS/MS parameters were set as follows: MS/MS resolution at 60,000; MS/MS AGC at 1e5; maximum Ion Time at 200 msec; Loop count at 15; Isowidth at 1.2 m/z; fixed first mass at 100 m/z; Normalised Collision Energy (NCE) at 32. The data dependent parameters were set as follows: min AGC at 2.4e3; Intensity Threshold at 1.2e4; Charge exclusion at unassigned 1, 7, 8; Peptide Match as Preferred; Exclude Isotope as On and Dynamic Exclusion at 45 sec. Based on these parameters the top 15 most abundant ions were analysed per instrument duty cycle. Data was acquired with Xcalibur software (Thermo Fisher Scientific).

#### Proteome and Phosphoproteome data analysis

The data processing was performed with COMPASS (Coon OMSSA (*Open Mass Spectrometry Search Algorithm*) *Proteomic Analysis Software Suite* [30] using DTA generation for data extraction from RAW files [31], and searching against the *T cruzi* Esmeraldo annotated protein database from TriTrypDB release 9.0 containing 358,801 entries, using OMSSA 2.1.8. The mass tolerance was set to 20 ppm for precursor ions and MS/MS tolerance was set to 0.8 Da. The enzyme was set to trypsin, allowing up to 3 missed cleavages. NEM on cysteine and TMT sixplex on lysine was set as a fixed modification. Acetylation of protein N-termini, deamidation of asparagine and glutamine, oxidation of methionine, pyro-glutamate and TMT sixplex on peptide N-termini were set as variable modifications. For analysis of the phospho-proteomic data, phosphorylation of serine, threonine and tyrosine were also set as variable modifications. Spectra were required to contain four or more multi-isotope peaks with the charge state derived from the input file. The false-discovery rate for protein and peptide level identification was set at 1%, using a target-decoy based strategy. TMT reporter intensities, corrected for reporter impurities, for each peptide were extracted using TagQuant 1.4 from within COMPASS. Protein groups were created using Protein Hoarder 2.4.7.0, also within COMPASS, ensuring quantified proteins were identified by 2 or more unique peptides. For proteome results Purity Corrected Normalised (PCN) values were used for fold change calculation (MTy/Ty). For phosphoproteome results, Purity Corrected (PC) values were normalized by the median between the total amount of proteins obtained from proteome data. PC manually normalized was used for phospho-fold change calculation (MTy/Ty). Statistical analysis for validation of the results was performed by T Student test (two-tail, 95%). Only peptides with ratio less than 0.8 or more than 1.2 (p-value less than 0.05) were considered.

#### *In silico* analysis of peptides and phosphopeptides

All the analysis on phosphopeptide, phosphorylation sites, number of phospho-residues were performed using filters tools in the *software Microsoft* ^*®*^*Office Excell*. The 303 phosphopeptides identified were used for kinase site prediction by the software GPS 2.1: enhanced prediction of kinase-specific phosphorylation sites, following the default parameters and high threshold. After kinase identification by the software, only sequences with a score above 20 were selected. For analysis of conservation between kinase family phospho-sites, all sequences obtained from GPS run were selected for sequence logo building for each kinase family, using the software *Weblogo* (http://weblogo.berkeley.edu/logo.cgi), following default parameters.

#### GO analysis

GO-terms enrichment analysis was performed by collecting the data information from version 40 of the TriTrypDB: product description and Computed/curated GO functions, Components and Process.

### Metabolomic analysis

#### Intracellular metabolites

The pellets were extracted by addition of 500 μL cold methanol:water (1:1, v/v) and lysed by sonication for 1 min at 30% frequency. Samples were centrifuged at 7,000 *x g* for 8 min. The supernatants were removed, from which 150 μL were submitted to the GC-MS derivatization protocol and 200 μL were collected and pooled with all other samples to obtain the quality control samples (QCs). A blank corresponds to the solvent extractor at the same extraction conditions. Samples, blank solution and quality controls were evaporated in a SpeedVac.

#### GC-MS derivatization

10 μL of O-methoxyamine (15 mg/mL in pyridine) were added to the samples, sonicated for 10 s and vortexed for 20 s. Samples were kept in the dark for 90 min at room temperature. Ten μL of BSTFA + 1% TMCS (v/v) were added to the extracts, homogenized and incubated for 30 min at 40°C. After this step, 100 μL heptane containing 10 mg/mL ^13^C-methyl-ester (internal standard) were added and the extract homogenized.

#### GC-MS equipment

GC-MS analyses were performed in a gas chromatography equipment (model 7890A, Agilent Technologies, CA, USA) coupled to a single quadrupole mass spectrometer detector (model 5975C inert XL, Agilent Technologies). The separation was carried out in a HP-5MS column (30 m length, 0.25 mm i.d., with 0.25 mm film composed of 95% dimethyl/ 5% diphenylpolysiloxane, Agilent Technologies). Helium was used as carrier gas at 1.0 mL/min flow rate. The GC injector was maintained at 250°C and samples were injected with 1:10 split, 10 mL/min of He was used. The temperature gradient was: initial oven temperature 60°C for 1 min, increased to 300°C at 10°C/min, following by cooling to 60°C in 1 min and maintained for 5 min. The run time was 25 min. The temperatures of the transfer line of the detector, filament source, and the quadrupole were maintained at 290, 230 and 150°C, respectively. The electron ionization source was -70 eV energy. The MS was operated in scan mode in the range 50–600 m/z. The software Mass Hunter B.07.01 (Agilent Technologies) was used for operation and data acquisition.

#### Data treatment and multivariate analysis

Raw data files were converted into *.mzData using the Qualitative Analysis Mass Hunter software B05.00 (Agilent Technologies). Previous processing by the XCMS package (version 1.24.1) was performed followed by running at R platform (3.1.0, R Foundation for Statistical Computing). The XCMS parameters were: fwhm (full width at half maximum of model peak) = 4, snthresh (signal-to-noise cutoff) = 1.5, max (maximum number of groups to identify in a single m/z slice) = 30, bw (bandwidth) = 2, and mzwid (width of overlapping m/z slices) = 0.25. Other parameters were kept at default values. After second grouping, fillPeaks was performed and the raw data was normalized by median and the intensity of the internal standard. Metabolites identification was assisted by Fiehn RTL Library (FiehnLib) and NIST MS, 2.0 g library (National Institute of Standards and Technology mass spectra library) after peak deconvolution with AMDIS 2.69 (Automated Mass spectral Deconvolution and Identification System) software. Peaks were assigned with basis on retention time (FiehnLib) and mass spectra fragmentation pattern (FiehnLib and NIST) after retention index and retention time analysis. To consider the identification match factor and NET factor should be greater than 700 and 85, respectively. Multivariate analyses PCA (Principal Component Analysis) and OPLS-DA (Orthogonal Partial Least Squares Discriminant Analysis) performed in SIMCA P+ software (12.0.1 version, Umetrics, CA, USA) were used to find differences between the studied groups. S-plot and Jack Knife from OPLS-DA were used to find the discriminant metabolites and T-Student test using Prism Graphpad (version 7) was applied to confirm the statistical significance (p-value < 0.05).

### Immunofluorescence

Trypomastigotes (ECM-treated for 120 min or control) were fixed in 2% paraformaldehyde for 15 minutes at room temperature, pelleted by centrifugation (4,000 *x g* for 5 minutes), washed twice in PBS, resuspended in PBS, added to a coverslip and dried at room temperature. After permeabilization of the parasites with PBS containing 1% BSA and 0.1% Triton X-100 for one hour at 37°C, anti-phosphoserine, anti-phosphothreonine, anti-phosphotyrosine (Invitrogen–dilution 1:200 for each antibody), anti-PAR monoclonal antibody (1:200) or anti–TcHexokinase (kindly provided by Dr. Ana Cáceres, Universidad de Los Andes, Venezuela) were added and incubated for 1 h at room temperature. After three washes with PBS containing 0.1% Triton X-100, the correspondent secondary antibodies were added (anti-rabbit or anti-mouse-Alexa 555 conjugated (1: 5000); followed by one hour incubation at 37°C. After three washes in PBS-0.1%-Triton X-100, the coverslips were faced under a solution containing 50% glycerol, 50% milliQ H_2_O 2 mM sodium azide, and 20 μg/mL of 4',6-diamidino-2-phenylindole, dilactate (DAPI-Invitrogen). The images were taken on an ExiBlue™ camera (Qimaging®) coupled to a Nikon Eclipse E 600 optical microscope and deconvoluted using the software Huygens Essential (Scientific Volume Imaging).

### Enzyme activities

Frozen pellets of trypomastigotes (1 x 10^9^ MTy or Ty) were resuspended in 1 mL of Lysis Buffer (30 mM Tris-HCl, pH 7.6, 1 mM EDTA, 0.1% Triton e 0.25 M sucrose, containing phosphatase and protease inhibitors, as described above, disrupted by ultrasonic for 4 x 10 s (frequency of 40%, Thomas GEX 600 apparatus). After centrifugation (10 000 *x g*, 15 min), the supernatant was separated and employed to measure the enzymatic activities (Hexokinase, Pyruvate kinase and Lactate dehydrogenase). In all cases, the amount of NADH / NADPH was measured spectrophotometrically at 340 nm and its concentration calculated (extinction coefficient = 6.220 M^-1^ cm^-1^) [32]. Three independent biological samples were employed. Due to the possible presence of ECM proteins in the MTy samples, the enzymatic activities were expressed by 1x10^8^ parasites. The number of parasites in each experimental point was also estimated by a calibration curve using the amount of paraflagellar rod protein in the Western blotting (anti-PAR monoclonal antibody 1:2000).

#### Hexokinase

Hexokinase activity was measured in the presence of a coupling system containing glucose-6-phosphate dehydrogenase. Increase in NADPH concentration was followed at 340 nm during 6 min at 30°C. 10 μl of the extract (1.10^8^ parasites) were incubated with the reaction mixture containing 20 mM triethanolamine buffer pH 7.2, 200 mM D-glucose, 0.80 mM ATP, 8 mM MgCl_2_, 1 mM NADP and 5 U glucose-6-phosphate dehydrogenase (Sigma®) [33]. As a control, the enzymatic activity was measured in the preparation incubated at 56°C for 1h. To measure HK activity in the extracts previously immunoprecipitated with anti-Hexokinase antibodies, parasite extracts (2.10^8^ parasites) were incubated under agitation with 3 μl of anti-Hexokinase antibodies and 50 μl of Protein A-Sepharose overnight at 4°C, washed extensively with PBS, treated or not with alkaline phosphatase as described below. The HK activity was measured directly in the pellet, as described herein.

#### Pyruvate kinase

Pyruvate kinase activity was measured in the presence of a coupling system containing lactate dehydrogenase for 6 min at 340 nm. Extracts of MTy or Ty were incubated in a mixture containing 38 mM potassium phosphate, pH 7.6, 0.43 mM phosphoenolpyruvate, 0.2 mM NADH (Sigma®), 6.7 mM MgSO_4_.7H_2_O (Sigma®), 1.3 mM adenosine diphosphate (ADP), 20 U lactate dehydrogenase and 1 mM fructose 1,6-diphosphate [34].

#### Lactate dehydrogenase

Lactate dehydrogenase activity was measured at 340 nm for 5 min at 30°C. Trypomastigotes extracts were incubated with the reaction mixture containing 50 mM potassium phosphate, pH 7.4, 0.2 mM NADH, 4 mM sodium pyruvate [32].

#### Alkaline phosphatase

Parasite extracts (3 x 10^8^ MTy or Ty) were incubated with 5 U of alkaline phosphatase (Boehringer Mannheim) for 1 h at 37°C, followed by measurement of hexokinase, pyruvate kinase or lactate dehydrogenase activities, as described above.

## Results and discussion

### Quantitative proteome and phosphoproteome of *T*. *cruzi* trypomastigotes

**Fig 1:**
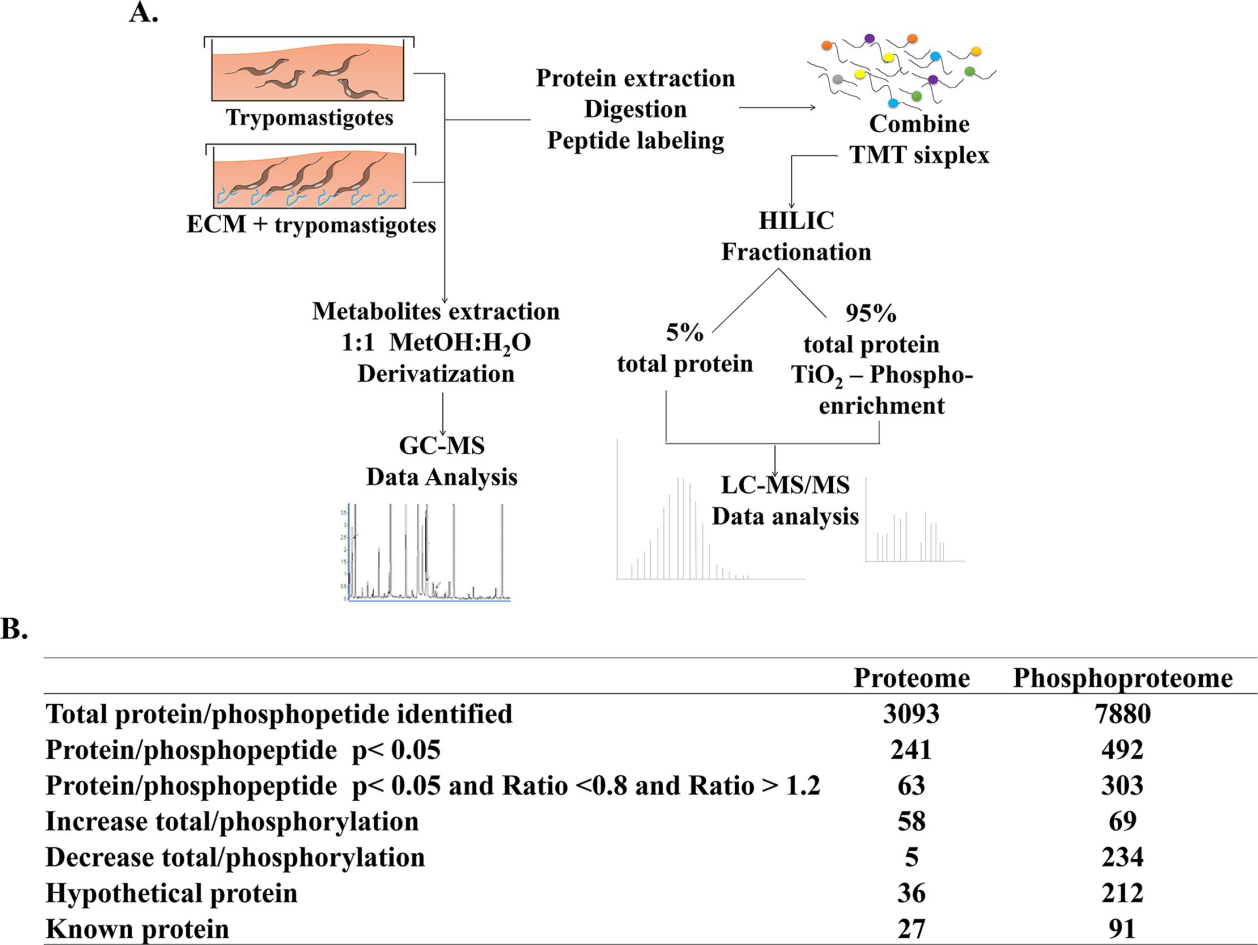
Protein and phosphoprotein changes in *T*. *cruzi* trypomastigotes following adhesion to ECM. (A) Schematic workflow of the experimental strategy to obtain proteome, phosphoproteome (LC-MS/MS) and metabolite quantification (GC-MS). (B) Overview of the number of proteins and phosphopeptides identified after LC-MS/MS.

Previous studies demonstrated that there is a significant decrease in protein phosphorylation upon parasite interaction with fibronectin or laminin, both components of the ECM. Thus we decided to investigate possible changes in protein phosphorylation that could occur upon trypomastigote interaction with the entire ECM. The experiments outlined in the scheme (Fig 1A) were performed in order to analyze both the proteome and the phosphoproteome of the parasites incubated with ECM.

The proteome and phosphoproteome analyses identified 3,093 proteins and 7,880 phosphopeptides, respectively, with FDR values less than 1% and p scores less than e-7 for peptide identification (Fig 1B). Thirty six proteins (57%) and 212 phosphopeptides (67%) correspond to proteins with unknown function (hypothetical proteins), as described by others in different trypanosomes [27, 35–42], which is in accordance with the number of proteins (49.2%) with unknown functions predicted by the genome sequence of *T*. *cruzi* [43] (Fig 1B). Sixty-three non-unique proteins from the proteome data exhibited significant variations, with only 5 showing reduction and 58 showing an increase in their protein level (Fig 1B, S1 Fig, S1 Table). Seventeen proteins showed changes where MTy/Ty ≥1.5, of which nine were hypothetical, including the one with the greatest change (MTy/Ty ≥12.9). Among the proteins with increased expression, it is worth emphasizing members of the gp85/trans-sialidase (TS) family, involved in host cell infection by *T*. *cruzi*, (MTy/Ty = 2.2 for one member of group II and Mty/Ty = 4.5 for one member of group IV); small GTP-binding protein rab6 (Mty/Ty = 3.8); ribosomal RNA processing protein 6 (MTy/Ty = 1.98; the splicing factor 3a; and the 2Fe-2S iron-sulfur cluster binding domain containing protein (Mty/Ty = 1.77).

**Fig 2:**
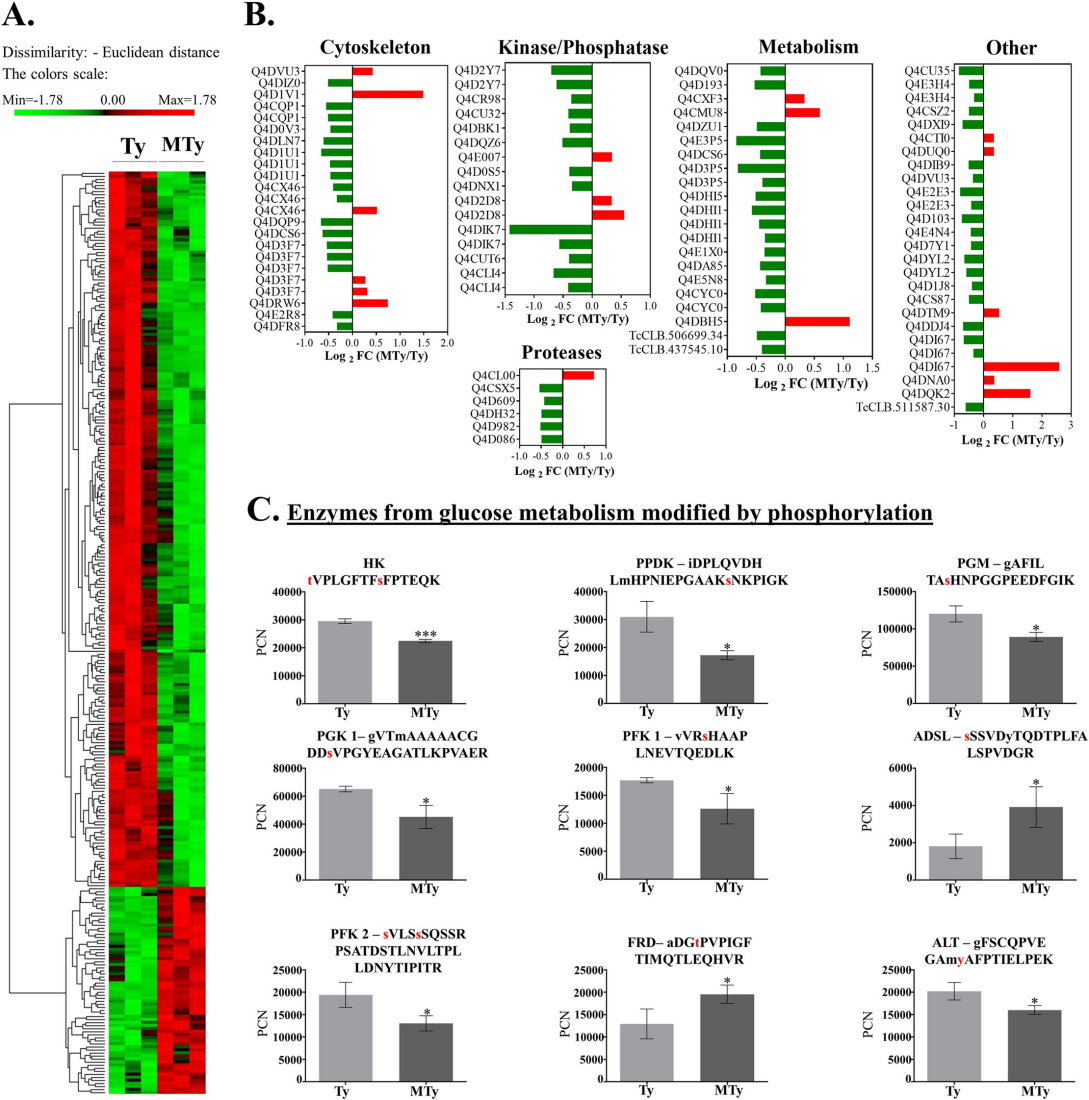
Phosphopeptides modified after trypomastigotes incubation with ECM for 120 min. (A) Heatmap with phosphopeptides (hypothetical and known proteins) distribution according to the PCN (normalized) intensities. (B) Functional phosphopeptide distribution of known proteins (only phosphopeptides with p-value < 0.05 and ratio (MTy/Ty) lower than 0.8 or higher than 1.2 were considered). (C) Quantification of the phosphopeptides (PCN—normalized) from enzymes involved in glucose metabolism. For proteins with more than one phosphosite, only the site with higher fold change (MTy/Ty) was shown, except for HK, for which the peptide with T and S phosphorylation was shown. HK- Hexokinase, PPDK- pyruvate phosphate dikinase, PGM- phosphoglycerate mutase, PGK- phosphoglycerate kinase, PFK1-phosphofructokinase 1, PFK2- phosphofructokinase 2, FRD- fumarate reductase, ADSL- adenylosuccinate lyase, ALT- alanine aminotransferase.

In the phosphoproteomic analysis, only phosphopeptides having MTy/Ty ratio below 0.8 or above 1.2, with p values less than 0.05, have been considered to evaluate the effect of ECM on trypomastigote protein phosphorylation. Among the 303 phosphopeptides selected by these criteria, 69 showed an increase and 234 a decrease in their phosphorylation levels. Of these, 91 (33%) are proteins with known function, of which 19 showed an increase and 72 a decrease in their phosphorylation levels (S2 Table; Fig 2A and 2B). Taken together the data indicate that adhesion of trypomastigotes to ECM overall leads to protein dephosphorylation, in agreement with previous observations with *T*. *cruzi* trypomastigotes incubated with ECM components, fibronectin and laminin [27].

Phosphorylation site analysis identified 371 differentially phosphorylated amino acid residues: 275 (74.1%) serine, 83 (22.4%) threonine and only 13 (3.5%) tyrosine residues, in accordance with the abundance of serine/threonine kinases found in *T*. *cruzi* [44], the fact that *T*. *cruzi* does not express receptor coupled tyrosine kinases but dual specificity kinases [44], as well as with the phosphoproteome data described for different stages of the parasite [42, 45, rev.^46^]. *In silico* analysis, as described below, suggested the involvement of different kinases (CAMK, TKL, CMGC, CK1, AGC and others).

To understand better the role of the proteins controlled by phosphorylation during the parasite response to the ECM, GO-terms enrichment analysis was performed. Additional information was obtained on the molecular function and/or sub-cellular localization of 29 proteins and 126 phosphopeptides previously labeled as hypothetical (unknown function) based on version 40 of the TriTrypDB. The data are shown in S1 Table and S2 Table. Most of proteins identified are related to structural function, pathogenicity, metabolism and protein phosphorylation. Both cytoplasm and axoneme seem to be the main localization of the identified phosphopeptides. Among the proteins identified, gp85/trans-sialidase (TS) family members, involved in infection of host-cells by *T*. *cruzi*, were enhanced in trypomastigotes incubated with ECM (MTy/Ty = 2.2 for one member of group II and MTy/Ty = 4.5 for one member of group IV). Small GTP-binding protein rab6 (MTy/Ty = 3.8), ribosomal RNA processing protein 6 (MTy/Ty = 1.98), splicing factor 3a and 2Fe-2S iron-sulfur cluster binding domain containing protein (MTy/Ty = 1.77) were also identified in the group of the proteins with an increased expression.

The following proteins are highly represented in the pool of proteins modified by phosphorylation (Fig 2B and S2 Table): proteins involved in metabolic processes (19 phosphopeptides); in phosphorylation/dephosphorylation (such as kinases and phosphatases, 20 phosphopeptides); structural proteins (such as flagellum and microtubule related proteins, 19 phosphopeptides); transport-associated proteins; and RNA/DNA binding elements. This suggests an extensive metabolic adaptation occurring in trypomastigotes prior to host-cell infection.

Of note, enzymes that participate in glucose metabolism, in addition to adenylosuccinate lyase (ADSL) and alanine aminotransferase (ALT) (Table 1; Fig 2C), lipid metabolism (3-oxo-5-alpha-steroid 4-dehydrogenase; putative (pseudogene) and ethanolamine phosphotransferase) (Fig 2B, S2 Table) are modified by phosphorylation.

Since a significant number of enzymes from the glucose metabolism were modified by phosphorylation, their role was further investigated.

**Table 1:** Identification of metabolic enzymes modified by phosphorylation after incubation of trypomastigotes with ECM for 120 min. Complete information for each phosphopeptide is shown in S2 Table.

Phosphopeptide	TriTryp ID	Protein Name	Ratio MTy/Ty	p-value	Phospho-Residue	Putative kinase	Putative phospho-peptide	Kinase Score
vVRsHAAPLNEVTQEDLK	TcCLB.506699.34	6-phospho-1-fructokinase (pseudogene); putative	0.711441246	0.033051	4S	Other/WEE/Myt1/PKMYT1	****VVRSHAAPLNE	5.583
sVLSSSQsSRPSATDSTLNVLTPLLDNYTIPITR	TcCLB.508569.130	6-phosphofructo-2-kinase/fructose-2;6-biphosphatase; putative	0.783549259	0.032736	8S	CAMK/CAMKL/QIK/SIK1	SVLSSSQSSRPSATD	4.074
rIEYLTEVYSTLSStCQSPGGPSDDEVTLGDVSR	TcCLB.508569.130	6-phosphofructo-2-kinase/fructose-2;6-biphosphatase; putative	0.730889744	0.006718	15T	CK1/VRK/VRK2	VYSTLSSTCQSPGGP	5.225
sVLSSSQsSRPSATDSTLNVLTPLLDNYTIPITR	TcCLB.508569.130	6-phosphofructo-2-kinase/fructose-2;6-biphosphatase; putative	0.783549259	0.032736	1S	TKL/STKR/STKR1/BMPR1B	*******SVLSSSQS	5.158
sVLSsSQSSRPSATDSTLNVLTPLLDNYTIPITR	TcCLB.508569.130	6-phosphofructo-2-kinase/fructose-2;6-biphosphatase; putative	0.67076967	0.028006	1S	TKL/STKR/STKR1/BMPR1B	*******SVLSSSQS	5.158
sVLSsSQSSRPSATDSTLNVLTPLLDNYTIPITR	TcCLB.508569.130	6-phosphofructo-2-kinase/fructose-2;6-biphosphatase; putative	0.67076967	0.028006	5S	TKL/STKR/STKR1/TGFbR1	***SVLSSSQSSRPS	5.333
sSSVDyTQDTPLFALSPVDGR	TcCLB.503855.30	adenylosuccinate lyase; putative (ADSL)	2.15819457	0.045825	6Y	TK/Src/SrcA/YES	**SSSVDYTQDTPLF	4.044
sSSVDyTQDTPLFALSPVDGR	TcCLB.503855.30	adenylosuccinate lyase; putative (ADSL)	2.15819457	0.045825	1S	TKL/STKR/STKR1/TGFbR1	*******SSSVDYTQ	5.333
gFSCQPVEGAmyAFPTIELPEK	TcCLB.506529.430	alanine aminotransferase; putative;	0.792580123	0.027477	12Y	TK/Eph/EphB1	QPVEGAMYAFPTIEL	10.317
tVPLGFTFsFPTEQK	TcCLB.508951.20	hexokinase; putative	0.760998922	0.000207	9S	CAMK/CAMKL/AMPK/AMPKA2	VPLGFTFSFPTEQK*	9.162
tVPLGFTFsFPTEQK	TcCLB.508951.20	hexokinase; putative	0.760998922	0.000207	1T	TKL/MLK/ILK/ILK	*******TVPLGFTF	3.003
tVPLGFtFSFPTEQK	TcCLB.508951.20	hexokinase; putative	0.566939414	0.02174	1T	TKL/MLK/ILK/ILK	*******TVPLGFTF	3.003
aDGtPVPIGFTIMQTLEQHVR	TcCLB.510215.10	NADH-dependent fumarate reductase; putative	1.514693923	0.041197	4T	CMGC/MAPK/JNK/JNK2	****ADGTPVPIGFT	31.509
gAFILTAsHNPGGPEEDFGIK	TcCLB.511911.130	phosphoglucomutase	0.743481685	0.012082	8S	CK1/VRK/VRK2	GAFILTASHNPGGPE	5.225
gVTmAAAAACGDDsVPGYEAGATLKPVAER	TcCLB.511419.40	phosphoglycerate kinase; putative	0.692608695	0.014714	14S	AGC/GRK/BARK/BARK1	AAACGDDSVPGYEAG	5.269
iDPLQVDHLmHPNIEPGAAKsNKPIGK	TcCLB.510101.140	pyruvate phosphate dikinase; putative	0.556518184	0.014326	21S	Other/PEK/HRI/EIF2AK1	IEPGAAKSNKPIGK*	4.658

* The asterisks indicate aminoacids not predictable by the GPS 3.0 software

### Phosphorylation of enzymes may regulate intermediate metabolism in *T*. *cruzi*

**Fig 3:**
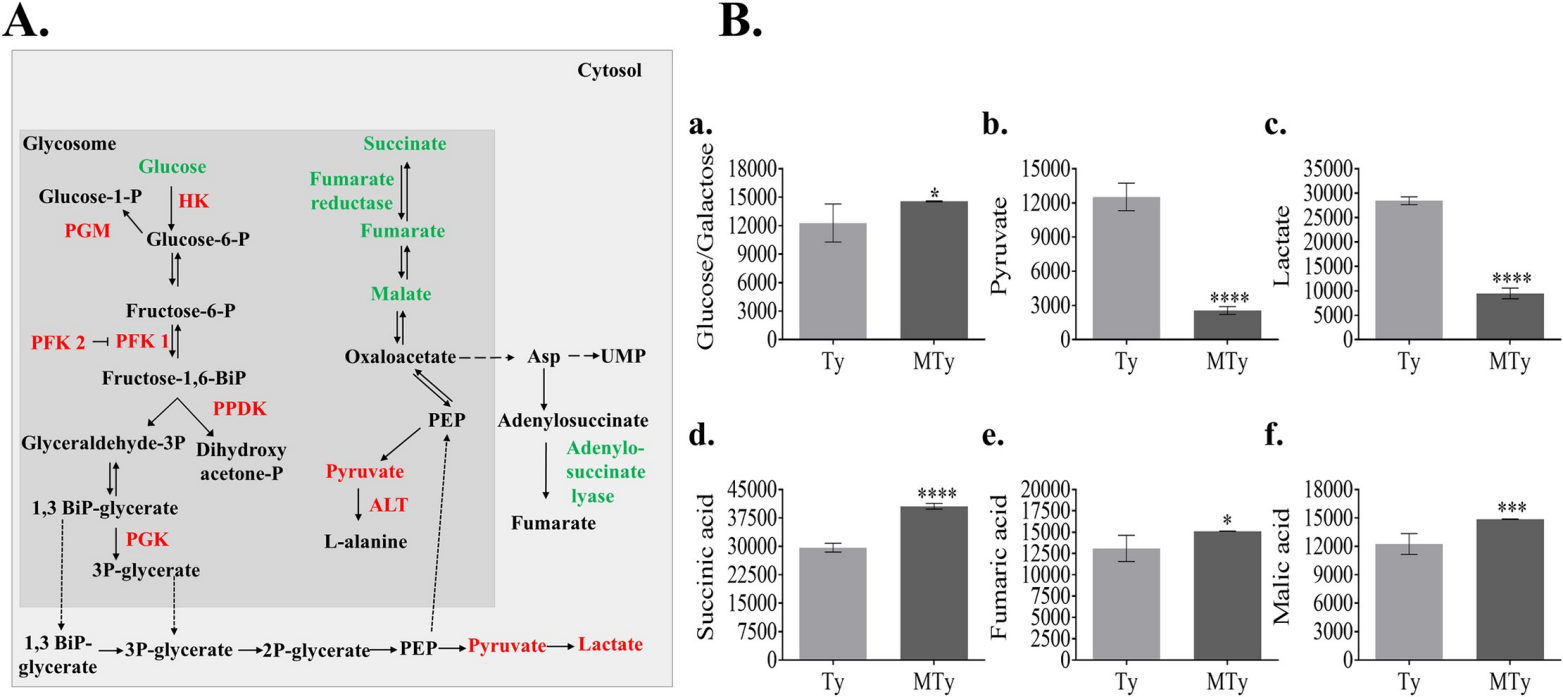
Metabolic enzymes modified by phosphorylation and metabolites quantification in trypomastigotes incubated with ECM. (A) Schematic representation of enzymes from the glycolytic and glycolytic branch modified by phosphorylation (green) or dephosphorylation (red) and metabolites that increase (green) or decrease (red) after trypomastigotes incubation with ECM for 120 min. (B) Relative abundance of metabolites in trypomastigotes (Ty) or trypomastigotes incubated with ECM (MTy). The abundance of the metabolite was normalized by the median and internal standard. The metabolites (a) Glucose/galactose; (b) Pyruvate; (c) Lactate; (d) Succinic acid; (e) Fumaric acid; f. Malic acid) are indicated in the graph. At least five distinct replicates were represented in each of the metabolite quantification. Asterisks indicate the significance of the difference between MTy and Ty samples, according to T- Student Test with p< 0.05.

Seven enzymes involved in carbohydrate metabolism were identified in the phosphoproteomic analysis: hexokinase (HK); 6-phosphofructo-2-kinase/fructose-2;6 bisphosphatase (PFK2); 6-phospho-1-fructokinase (PFK1)(pseudogene); phosphoglucomutase (PGM); pyruvate phosphate dikinase (PPDK); phosphoglycerate kinase (PGK) and NADH-dependent fumarate reductase (FRD), in addition to adenylosuccinate lyase and alanine aminotransferase (Fig 2C, Fig 3, Table 1, S2 Table). Except for NADH-dependent fumarate reductase (MTy/Ty = 1.5) and adenylosuccinate lyase (MTy/Ty = 2.2), all the others showed decrease in their phosphorylation levels when trypomastigotes were incubated with ECM (Table 1 and Fig 3). Most of these enzymes are localized in the glycosomes, a peroxisome-like organelle essential for trypanosomatids survival and characterized for containing most of the glycolytic/gluconeogenic pathways in kinetoplastids, in addition to enzymes of other metabolic pathways, such as the pentose phosphate pathway, beta-oxidation of fatty acids, and biosynthesis of pyrimidines (rev. [47–49]). Likewise, phosphorylation of enzymes involved in carbohydrate metabolism was described in the proteome and phosphoproteome of the glycosomes in *T*. *brucei* and *Leishmania donovani* [40, 41, 50].

Since the data suggested possible changes in the metabolism of *T*. *cruzi*, the metabolite content was analyzed by GC/MS to understand better the response of trypomastigotes upon adhesion to ECM.

### Metabolite quantification by GC-MS analysis suggests broader metabolic changes in trypomastigotes incubated with ECM

GC-MS analysis allowed the identification of 21 metabolites with a significant variation (p < 0.05) in the MTy/Ty ratio (S5 Table), from which significant changes were found for carbohydrate, lipid and amino acid metabolites. Some of these metabolites are substrates or products of the enzymes modified by phosphorylation that are found, although not exclusively, inside the glycosomes (Fig 3A and 3B, Table 1). The following metabolites from the glycolytic pathway were modified in parasites upon incubation with ECM: increase in glucose/galactose (molecules indistinguishable in the GC-MS methodology) with ECM (MTy/Ty = 1.2); decrease in pyruvic acid (MTy/Ty = 0.2) and lactic acid (MTy/Ty = 0.34). Interestingly, metabolites derived from the glycolytic pathway branch and common to TCA cycle: succinic acid (MTy/Ty = 1.37), malic acid (MTy/Ty = 1.21) and fumaric acid (MTy/Ty = 1.15), were increased in parasites incubated with ECM. Succinate is considered the main source of reducing equivalents to the respiratory chain through the action of a NADH-dependent fumarate reductase and it is also (in addition to alanine) one of the main products excreted by trypanosomatids [48, 51], although lactate excretion by *T*. *cruzi* [52] may increase, depending on metabolic adaptations. These changes in metabolite level do not appear to correlate with changes in metabolic enzyme levels (S1 Table). Rather, they may reflect modulation of metabolic enzyme activity. Although less representative in the metabolite quantification, an increase in free amino acids in MTy (tyrosine, glycine or isoleucine, MTy/Ty ratio ≈1.4) and fatty acids (mainly palmitic acid, MTy/Ty ratio ≈1.4) may also indicate wider changes in the metabolism of MTy (S5 Table).

### Phosphorylation level of hexokinase, pyruvate kinase and lactate dehydrogenase-like may contribute to the control of glucose metabolism in trypomastigotes incubated with ECM

To analyze the potential role of metabolic enzyme phosphorylation in modulating the glycolytic pathway in MTy, enzymatic activities of hexokinase/glucokinase (HK/GK) and pyruvate kinase (PK) were determined, as well as for lactate dehydrogenase (LDH). Although PK and LDH were not detected in our phosphoproteomic analysis (S2 Table), these enzymes were included because significant depletion of pyruvate and lactate were observed in MTy (S5 Table). Further, as pointed out before, the changes in the metabolite levels seem to be independent of the relevant metabolic enzyme expression levels accordingly to the proteomic data (S1 Table).

The enzymes HK and GK catalyze the formation of glucose-6-phosphate, the first reaction of the glycolytic pathway and both lack the regulatory allosteric inhibition by glucose-6-phosphate, common to other organisms. Both are localized inside the glycosomes, of which HK presents the highest activity [53, 54]. A distinct HK, which phosphorylates glucose and fructose, as well as GK activity were also described in the cytosol [53]. The affinity of HK for glucose is higher than that of GK (Km values of 0.06 mM and 0.7 mM, respectively) in addition to the higher amounts of HK over GK in the parasite [55]. However, in the present work we could not separate the activities of both enzymes and, thus, they are collectively represented by HK/GK activities.

**Fig 4:**
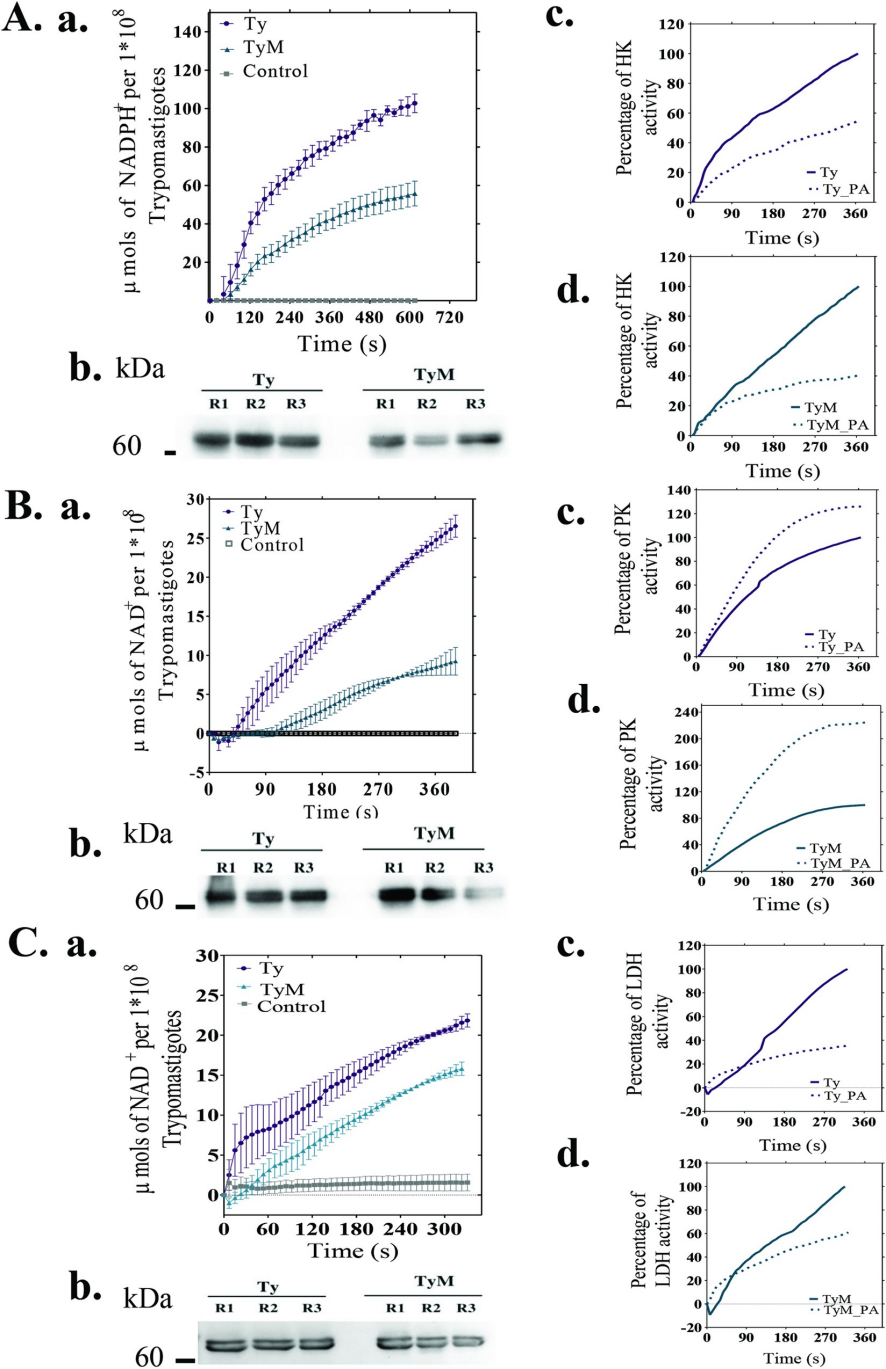
**Enzymatic activity of three enzymes from the glucose metabolism: HK (A) PK (B) and LDH (C).** (a) Quantification of the respective enzyme activity in extracts of trypomastigotes of *T*. *cruzi* incubated (MTy) or not (Ty) with ECM and expressed for 1 x 10^8^ parasites. (b) Loading control of PAR (*Paraflagellar-rod proteins*) for the three replicates used in the measure of each enzyme activity: HK (A), PK (B) and LHD (C). (c) Quantification of the enzyme activity of extracts from trypomastigotes incubated (Ty_AP) or not (Ty) with alkaline phosphatase. (d) Relative quantification of enzyme activity of extracts of trypomastigotes incubated with ECM followed by incubation (MTy_AP) or control (Ty) with alkaline phosphatase and expressed as percentage of activity. The number of parasites (Fig 4a) was based on the calibration curve presented in S3 Fig.

The three phosphorylated residues of *T*. *cruzi* hexokinase (S161, T153, T159) (Table 2, S1 Table, S2 Fig) were located in the same peptide identified by LC-MS/MS and presented similar changes in the phosphorylation level (MTy/Ty ratio = 0.76; 0.76 and 0.57, respectively). The identified phosphorylated peptide is localized in the catalytic domain or in the substrate-binding site of the enzyme, according to the alignment of *T*. *cruzi*-HK (TcCLB.508951.20), *T*. *brucei* (Tb927.10.2010) and the three isoforms of human-HK (P52790; P19367; P52789), performed by ClustalW platform (S2B Fig). HK/GK activities from MTy and Ty homogenates were measured spectrophotometrically in the presence of a coupling system containing glucose-6-phosphate dehydrogenase. HK/GK activity is clearly reduced (by approximately 46%) in MTy relative to Ty (Fig 4A,a). Previous treatment of Ty and MTy homogenates with alkaline phosphatase significantly reduced the activity (approximately 45% for Ty and 59% for MTy extracts, Fig 4A,c,d). Also, the activity was drastically reduced (approximately 70%) when the homogenate was previously treated at 56°C for 1 h. Since HK is inhibited by small phosphate molecules, such as PPi present in distinct organelles including glycosomes [53, 56, 57], the experiment was repeated with the parasite extract previously immunoprecipitated with anti-HK antibodies. Similar results have been obtained, confirming the relevance of phosphorylation for HK/GK activity (S4 Fig). Inhibition of the enzymatic activity by dephosphorylation is consistent with the accumulation of glucose detected in MTy. However, one cannot rule out other possibilities, such as changes in glucose transport in MTy, alterations of HK oligomerization, which is usually tetrameric [54] or somehow by contributing to the hysteretic and cooperative behavior of HK at low enzyme concentration described in *T*. *cruzi* epimastigotes [58]. The phosphorylation of HK could be by auto-phosphorylation [59] or by protein kinase(s). Of note, the same pattern of HK in MTy and Ty inside the glycosomes was shown by immunofluorescence using specific anti-HK antibodies (S2A Fig). These data also indicate that no significant changes in the number of glycosomes occur in MTy, in accordance with the literature, where approximately the same number was found in the different forms of *T*. *cruzi*, in contrast to the variability described during the life cycle of other species [60].

**Table 2:** Identification of kinases and phosphatases modified by phosphorylation after incubation of trypomastigotes with ECM for 120 min. Complete information is shown in S2 Table.

Phosphopeptide	TriTryp ID	Protein Name	Ratio MTy/Ty	p-value	Phospho-Residue	Putative kinase	Putative phospho-peptide	Kinase Score
iSTGGKDEPALVSLVLAGDFNsDAGSPPIR	TcCLB.504073.10	endonuclease/exonuclease/phosphatase; putative	0.779478808	0.000646	22S	CAMK/CAMKL/QIK/SIK2	VLAGDFNSDAGSPPI	7.514
kLSPSEPNVAyICSR	TcCLB.507993.80	glycogen synthase kinase 3; putative	0.779756115	0.042338	11Y	TK/Tec/ITK	PSEPNVAYICSR***	2.44
aFVPLIPVAQNANPTPESPFLLsPEKDR	TcCLB.504213.90	inositol polyphosphate kinase-like protein; putative	0.674636314	0.034507	23S	AGC/GRK/BARK/BARK1	PESPFLLSPEKDR**	5.269
aFVPLIPVAQNANPTPESPFLLsPEKDR	TcCLB.504213.90	inositol polyphosphate kinase-like protein; putative	0.372851136	0.006706	23S	AGC/GRK/BARK/BARK1	PESPFLLSPEKDR**	5.269
eDTQDQNKtHyVTHR	TcCLB.511573.40	mitogen-activated protein kinase; putative	0.752549702	0.031487	9T	STE/STE7/MEK3/MAP2K3	DTQDQNKTHYVTHR*	4.309
eDTQDQNKtHyVTHR	TcCLB.511573.40	mitogen-activated protein kinase; putative	0.752549702	0.031487	11Y	TK/Src/SRM/PTK6	QDQNKTHYVTHR***	4.177
nTFELTNGTPTIAGVSFVSNSsPTGGAPSFFR	TcCLB.510105.130	phosphatidylinositol-4-phosphate 5-kinase type II beta; putative	0.711653701	0.001718	22S	Other/PLK/SAK/PLK4	VSFVSNSSPTGGAPS	4.9
tFtLCGTPEYLAPEVIQSR	TcCLB.509805.10	protein kinase A catalytic subunit; putative	1.269816312	0.038173	3T	Other/CAMKK/Meta/CaMKK2	*****TFTLCGTPEY	7.822
tFtLCGTPEYLAPEVIQSR	TcCLB.509805.10	protein kinase A catalytic subunit; putative	1.269816312	0.038173	1T	STE/STE20/TAO/TAOK1	*******TFTLCGTP	9
aAEVSVGEsNTPANTPNNSR	TcCLB.503757.40	protein kinase ck2 regulatory subunit; putative	0.758482269	0.049888	9S	AGC/GRK	AEVSVGESNTPANTP	7.991
qAGVGNPPPLSPLQALCSPtATGLSPVLLGEKGDHHLPVSK	TcCLB.509231.20	protein kinase; putative	0.760798278	0.04098	20T	CMGC/DYRK/DYRK1/DYRK1B	LQALCSPTATGLSPV	7.575
tTVASAATAVASVTsPPLLSSAVSSK	TcCLB.509099.150	protein kinase; putative	0.784036858	0.010475	15S	CMGC/MAPK/JNK/JNK3	TAVASVTSPPLLSSA	11.042
rVLtPSGGFDDTYSSGIELFDEIHR	TcCLB.510257.130	protein kinase; putative	0.764883202	0.007529	4T	CMGC/MAPK/p38/MAPK13	****RVLTPSGGFDD	4.706
iIDFGSSCYLTDNLSsYVQSR	TcCLB.506869.60	protein kinase; putative	1.468291844	0.04288	16S	STE/STE20/FRAY/OSR1	YLTDNLSSYVQSR**	1.307
iIDFGSSCYLTDNLsSYVQSR	TcCLB.506869.60	protein kinase; putative	1.261821895	0.044097	15S	STE/STE7/MEK3/MAP2K4	CYLTDNLSSYVQSR*	1.325
tTVASAATAVASVTsPPLLSSAVSSK	TcCLB.509099.150	protein kinase; putative	0.784036858	0.010475	1T	TKL/MLK/MLK/ZAK	*******TTVASAAT	9.25
iGLGGIGTFTSSsSSPK	TcCLB.510089.130	protein kinase; putative	0.70009069	0.028877	13S	TKL/STKR/STKR1/TGFbR1	IGTFTSSSSSPK***	5.333
gGHPLHQENQmsEEDEDVEALPHSVSQR	TcCLB.484949.9	serine/threonine protein kinase; putative	0.629335525	0.0197	12S	CMGC/CK2	LHQENQMSEEDEDVE	9.894
tPETTLGGVLAEVAPsLISHSFPLELGESqTAAHQELHPDLGR	TcCLB.484949.9	serine/threonine protein kinase; putative	0.750070579	0.029801	1T	CMGC/MAPK/p38/MAPK13	*******TPETTLGG	4.706
tPETTLGGVLAEVAPsLISHSFPLELGESqTAAHQELHPDLGR	TcCLB.484949.9	serine/threonine protein kinase; putative	0.750070579	0.029801	16S	Other/NEK/NEK9/NEK9	VLAEVAPSLISHSFP	7.983
lLLHPSHnGAAALASASIEsPVGR	TcCLB.507757.50	serine/threonine-protein phosphatase PP1; putative	0.65305933	0.024747	20S	STE/STE11/MEKK1/MAP3K1	LASASIESPVGR***	3.517
lLLHPSHNGAAALAsASIESPVGR	TcCLB.507757.50	serine/threonine-protein phosphatase PP1; putative	0.61302874	0.017711	15S	STE/STE20/KHS/HPK1	NGAAALASASIESPV	5.683

* The asterisks indicate aminoacids not predictable by the GPS 3.0 software

Pyruvate kinase (PK) was not detected in the phosphoproteome/proteome described herein, as pointed out above, but the low amount of pyruvate found in MTy led us to measure a corresponding enzymatic activity. In the cytosol, PK catalyzes the formation of pyruvate and ATP from phosphoenolpyruvate and ADP and, in the case of *T*. *cruzi* epimastigotes, PK is inhibited by millimolar concentrations of ATP and Pi and activated by micromolar concentrations of fructose 2,6-bisphosphate (rev. [48, 61]) by the tetrameric stabilization of PK in response to the effector binding [62].

As shown (Fig 4B,a), PK activity is strongly inhibited in trypomastigotes incubated with ECM (approximately 65%). Treatment of the enzyme with alkaline phosphatase increased its activity, mainly in MTy homogenates (25% Ty and 125% MTy), as shown in Fig 4B,c,d. The decrease in pyruvate content observed could be attributed to the inhibition of pyruvate kinase in the cytosol and/or pyruvate phosphate dikinase in the glycosome, which would lead to an increase of dicarboxylic acids from the glycolytic branch (succinate, fumarate and malate) in the glycosome, (cf. Fig 3 and S5 Table). However, higher consumption of pyruvate, for example by its conversion to acetyl-CoA inside the mitochondria or to lactate in the cytosol, cannot be ruled out.

Lactate dehydrogenase-like (LDH) activity was measured in the parasite homogenate due to the decrease observed in lactate content in MTy (S5 Table). In many organisms, a tetrameric form of the enzyme catalyzes the oxidation of lactate to pyruvate in the presence of NAD^+^ as hydrogen acceptor. LDH activity in *T*. *cruzi* epimastigotes was attributed to the isoenzyme I of ∂-hydroxyacid dehydrogenase localized inside the glycosomes and in the cytoplasm [63] and to an unknown protein in *T*. *brucei* [51], since a typical lactate dehydrogenase is absent from the genomes of trypanosomatids. The measurement of LDH activity showed approximately 33% reduction in MTy in comparison to trypomastigotes (Fig 4C,a), in agreement with the decrease of lactate detected in MTy. LDH was also inhibited by alkaline phosphatase treatment (approximately 65% for Ty and 41% for MTy extracts), reinforcing the role of phosphorylation in modulating LDH activity (Fig 4C,c,d).

Alanine, rather than lactate, is usually the main product of the reduction of pyruvate in *T*. *cruzi*, a reaction catalyzed by alanine aminotransferase inside the glycosomes (rev. [48, 49]). Lactate excretion by the parasite [52] may increase depending on metabolic adaptations, as described for the procyclic forms of *T*. *brucei* [51] and may explain the aforementioned results in trypomastigotes. Although no significant differences in alanine content between MTy and Ty were detected by GC/MS analysis, alanine aminotransferase is less phosphorylated in trypomastigotes incubated with ECM and may be responsible for the switch to the LDH reaction for NADH-reoxidation. Since *T*. *cruzi* possesses the enzyme repertoire for gluconeogenesis, this pathway may also be activated in MTy, resulting in higher consumption of pyruvate, lactate and glycerol, although no reserve polysaccharide was detected and gluconeogenesis has not been fully established in *T*. *cruzi*.

### Analysis of putative kinases involved in trypomastigote response to ECM

Our data suggest that incubation of trypomastigotes with ECM triggers metabolic adaptations in the parasites, and that phosphorylation, or more specifically protein dephosphorylation, may be involved in these processes. In spite of the relevance of the dephosphorylation and the high representative number of phosphatases in the genome and proteome of *T*. *cruzi* [64, 42], only two protein phosphatases with diminished phosphorylation levels in MTy were found: an endonuclease/exonuclease/phosphatase responsible for dephosphorylation of DNA sequences (TcCLB.504073.10) and PP1, a serine/threonine phosphatase (TcCLB.507757.50). Serine/threonine phosphatases are abundant in *T*. *cruzi* and constitute more than 50% of the 86 protein phosphatases in the genome. Protein phosphatase PP1 is dephosphorylated in MTy (MTy/Ty = 0.65), which would be expected to increase its enzymatic activity and consequently contribute to the dephosphorylation of proteins [65] (Table 2; S2 Table). Other protein phosphatases, such PP2A, PP2C or dual specific phosphatase were described in the proteome / phosphoproteome of *T*. *cruzi* by different groups or in the glycosomes of *T*. *brucei* [41] or *L*. *mexicana* [50], which might also contribute to the decrease of the phosphorylation level of the proteins.

**Fig 5:**
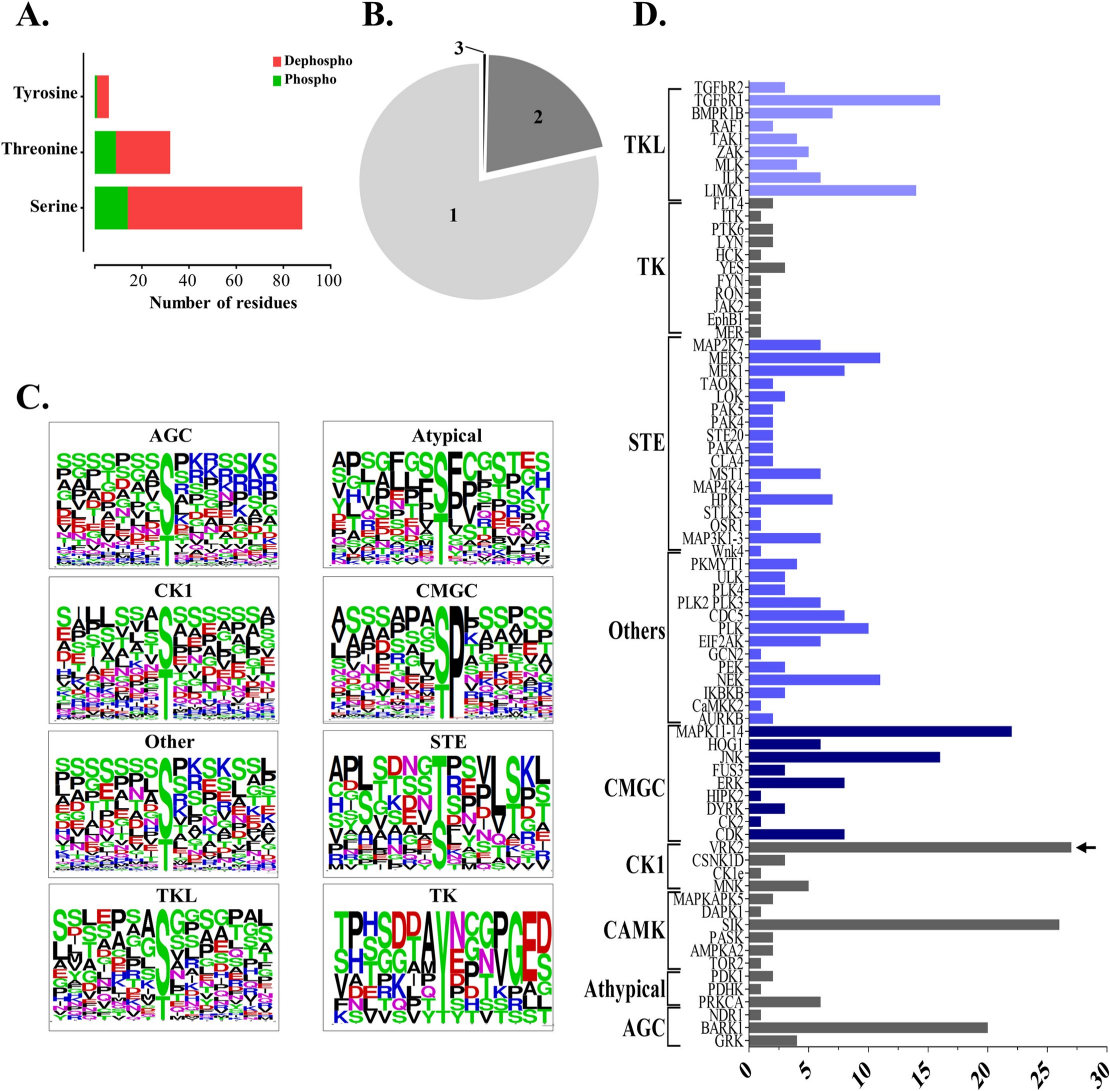
Conservation of the phosphorylation sites among the modified phosphopeptides in trypomastigotes incubated with ECM and characterization of the putative kinases involved in the phosphorylation events. (A) Number of amino acid residues (S, T and Y) modified by phosphorylation (green) or dephosphorylation (red) in ECM-treated trypomastigotes. (B) Number of phosphorylated amino acids residues for each phosphopeptide. (C) Sequence logo of the peptides identified by the software GPS 2.1 distributed according to the kinase family. (D) Identification and quantification of putative kinases responsible for phosphorylation of the phosphopeptides modified after parasite-ECM incubation. Only phosphosites with higher score were selected. The prediction was made using the software GPS 2.1: enhanced prediction of kinase-specific phosphorylation sites. The arrow indicates the kinase family able to phosphorylate the majority of peptides of the phosphoproteome.

To understand further the signaling pathways activated in the parasite upon contact with host ECM, the kinases capable of phosphorylate the peptides detected by LC-MS/MS were predicted by the GPS 2.1 software. Only the higher scores for each phospho-residue (S, T and Y) in the peptide were selected, totaling 378 putative kinases for the 303 phosphopeptides identified by LC-MS/MS (S3 and S4 Tables). Most of the identified sites correspond to modifications by serine/threonine kinases and the phosphorylation of tyrosine was attributed to the dual-specificity kinases, in agreement with the absence of conventional tyrosine kinases in the genome of trypanosomatids [44]. Of the 303 phosphopeptides analyzed, 78.5% and 21.1%, respectively, presented one or two phosphorylated sites and only one phosphopeptide showed three phosphorylated-sites (Fig 5A and 5B). The phosphorylated sites identified herein are predicted to be modified mainly by elements of the CMGC kinases superfamily (68), STE kinases (62), TKL kinase (61), AGC kinase (31), CK1 family (31) (Fig 5D, Tables 1 and 2, S3 and S4 Tables). Kinases that do not belong to any characterized family are grouped as “Others”.

Only the catalytic subunit of protein kinase A, a member of the AGC kinase superfamily showed in MTy a slightly increase in its phosphorylation level (MTy/Ty = 1.27). The phosphorylation of T197 of mouse PKA, located in the activation loop, which corresponds to T35 described herein for *T*. *cruzi*, increases PKA activity, indicating that upon interaction with ECM, PKA is activated [66, 67]. Proteins responsible for a plethora of biological phenomena in *T*. *cruzi* have been described as PKA substrates, such as kinases (type III PI3 kinase-Vps34, PI3 kinase, mitogen-activated extracellular signal-regulated kinase), cAMP-specific phosphodiesterase (PDEC2), putative ATPase, DNA excision repair protein, aquaporin, hexokinase and members of gp85/TS [68, 69]. The role of PKA during metacyclogenesis (differentiation of epimastigotes to metacyclic trypomastigotes [70]) or during the amastigogenesis (differentiation of trypomastigotes into amastigotes [42]) is well established, with the stimulation of adenylyl cyclase and increment in cAMP concentration during the process. Albeit the relevance of PKA in the physiology of the parasite, their specific role in trypomastigotes incubated with ECM was not determined.

In contrast to PKA, reduction in phosphorylation was detected in the majority of the kinases, for example in glycogen synthase kinase 3 (GSK3) and ERK1/2, presumably leading to a decrease of their activities in MTy: Y187 from GSK3 (MTy/Ty = 0.78) corresponds to human Y216 also located in the activation loop and whose phosphorylation is necessary for activity [71]; T190 and Y192 from mitogen-activated protein kinase (MTy/Ty = 0.75), corresponds to human T185/Y187, whose phosphorylation is necessary for ERK1/2 activation [72]. Dephosphorylation of ERK1/ 2 was also observed in the incubation of trypomastigotes with laminin or fibronectin [27].

Decrease in phosphorylation of other kinases was also found: protein kinase ck2 regulatory subunit (MTy/Ty = 0.76), inositol-related signaling kinases; inositol polyphosphate kinase-like protein (MTy/Ty = 0.67) and phosphatidylinositol-4-phosphate 5-kinase type II beta (MTy/Ty = 0.37) (Table 2). Whether this is a reflection of their activation status remains to be determined.

The phosphopeptides assigned to particular kinase families by the GPS 2.1 software (Fig 3D) were used for the construction of sequence logos, which correspond to sequence alignment with the central point as the likely phospho-amino acid residue (Fig 3C). For the AGC kinase families, "other" and CK1, no consensus was observed in the amino acid sequences surrounding the S/T residue. For the "Atypical", TK and STE families some amino acids were identified with a high level of conservation. The analysis of the sequence logos (Fig 3C) indicates some conservation relative to the sites already characterized for humans, such as the group of peptides phosphorylated by CMGC, where a proline next to the phosphorylation site was also identified; lysine and arginine near the phosphorylation site for AGC; residues of aspartate and glutamate for CK1 family substrates and the conservation of phenylalanine and proline residues near the phosphorylated site for the atypical kinases.

### Concluding remarks

Incubation of *T*. *cruzi* trypomastigotes with the extracellular matrix results in important and more extensive changes than the ones previously described by the incubation of trypomastigotes with fibronectin or laminin [27]. Reduction in the phosphorylation level of proteins seems to be a general event in trypomastigotes incubated with ECM (Tables 1 and 2, S2 Table). Kinases, except for PKA, PP1 phosphatase and enzymes from the glycolytic pathway and probably the glycolytic branch exemplify these decreases (S2 and S3 Tables). Strikingly, correlating with the observed decrease in phosphorylation level of the enzymes, a significant inhibition of hexokinase, pyruvate kinase and lactate dehydrogenase-like were detected. (S2 and S5 Tables, Fig 4). These results, in association to the slightly increase of glucose and drastic reduction of pyruvate and lactate strongly suggest that ECM triggers important reduction in the glycolytic pathway in *T*. *cruzi* trypomastigotes. Although hexokinase is among the many substrates described for PKA [69], a possible correlation between these enzymes has not been explored herein. Interestingly, trans-sialidases, surface glycoproteins belonging to the *T*. *cruzi* gp85/trans-sialidase family, are also one of the substrates for PKA [69] and PKA activity and trans-sialidase expression has been associated with differentiation and invasion of host cells by *T*. *cruzi* [73]. A similar coincidence of increase in PKA activity and expression of two members of the gp85/trans-sialidase family were observed upon incubation of trypomastigotes with ECM (MTy/Ty = 2.2 and 4.5, S1 Table), perhaps preparing the parasites for an efficient invasion of the host cell. The possibility that members of the large gp85/ trans-sialidase family interact with different components of ECM to trigger all the modifications described here remains to be determined.

Taken together, the data presented herein suggest reprogramming of the metabolism of trypomastigotes triggered by their interaction with the extracellular matrix, an obligatory step before cell invasion and differentiation into amastigotes, the multiplicative stage of *T*. *cruzi* in the vertebrate host. The reduction in glycolytic enzyme activity in trypomastigotes by phosphorylation/dephosphorylation events seems to be part of this reprogramming, with the involvement of yet to be identified protein kinases and phosphatases.

## Supporting information

S1 Fig Proteome and phosphoproteome profile. A. Volcano plot of total protein identified after LC-MS/MS. The Y-axis represents the -log 2 p-value (T-test) and X-axis represents the -log 2 Ratio of Protein intensity MTy/Ty. B. Volcano plot of total phosphopeptides identified after phospho-enrichment followed by LC-MS/MS. Y and X-axis are the same as represented in A. C. Heat map of the 64 proteins with significant differences between MTy and Ty samples. The Tritryp ID for each protein identified is indicated on the right.
(TIF)

S2 Fig HK characterization in trypomastigotes incubated (MTy) or not (Ty) with ECM. (A) Sub-cellular localization of Hexokinase (red), PFR (green) and nuclei (blue) in trypomastigotes. (B) Sequence aligment between isoforms of human-HKs (P52790; P19367; P52789) and Tc-HK (Q4D3P5/TcCLB.508951.20). The arrows indicate the phospho-residues and orange box indicates the phosphopeptide identified after phosphoproteome analysis.
(TIF)

S3 Fig **Correlation between the number of trypomastigotes and PFR loading for Ty and MTy extracts used for HK (A), PK (B) and LDH (C) enzymatic quantification assays.** (a) Immunoblotting of 20 x105 to 1.2 x 105 trypomastigotes extracts with antibody anti-Paraflagellar rod proteins (PFR). (b) Curve of linear correlation between curve area of the immunoblotting bands (a) and trypomastigote numbers. (c) Estimative of parasite number for each extract employed for enzymatic quantification assay shown in Fig 4 and S4 Fig.
(TIFF)

S4 Fig Hexokinase activity in Ty and MTy extracts immunoprecipitated with anti-Hexokinase antibodies (HK IP) and treated with alkaline phosphatase (AP). Extracts from parasites previously incubated with ECM for 2h (TyM2h) or with medium (Ty2hC, control) were immunoprecipitated with anti-HK antibodies (TyMHK IP and Ty2hC+), treated (+AP) or not with AP, followed by the measurement of HK activity. C- Ty extract. The number of parasites was based on the calibration curve presented in S Fig 3.
(TIFF)

S1 Table Proteome overview. Proteins identified with significative difference between Ty and MTy (T-Student Test, p < 0.05 for TMT normalized quantification (PCN)). *p-Scores* represent the confidence of protein identification by the software. Only proteins with *p-score* < e-7 were selected.
(XLSX)

S2 Table Phosphoproteome overview. Phosphopeptides identified with significant differences between Ty and MTy extracts (T-Student Test, p < 0.05 for TMT normalized quantification (PCN manual values)). *p-Scores* represent the confidence of protein identification by the software. Only p-score < e-7. Residues of S, R and Y represented in lower case correspond to the phosphorylation sites.
(XLSX)

S3 Table Phosphoproteome and identification of putative kinases using the GPS analysis. Phosphopeptides identified with significant differences between Ty and MTy extracts (T-Student Test, p < 0.05 for TMT normalized quantification (PCN manual values)). Putative kinase family able to phosphorylate each one of the substrates and the peptide sequence surrounding the phosphorylation site, are represented in the Table. The score calculated by GPS algorithm evaluates the potential of the phosphorylation.
(XLS)

S4 Table Phosphoproteome and identification of only one putative kinase (upper score, after GPS analysis) for each phosphopeptide substrate. Phosphopeptides identified with significant differences between Ty and MTy extracts (T-Student Test, p < 0.05 for TMT normalized quantification (PCN manual values)). The putative kinase family able to phosphorylate each substrate and the peptide sequence surrounding the phosphorylation site are represented. Only the upper score calculated by GPS algorithm for each phosphopeptide was selected.
(XLSX)

S5 Table Quantification of metabolites in trypomastigotes incubated (MTy) or not (Ty) with ECM for 120 min. (XLSX)
