# Supplementary figures and images for: Reprogramming of Trypanosoma cruzi metabolism triggered by parasite interaction with the host cell extracellular matrix
Source: PLoS Negl Trop Dis. 2019 Feb 6;13(2):e0007103. doi: 10.1371/journal.pntd.0007103 (PMC6380580; doi:10.1371/journal.pntd.0007103)

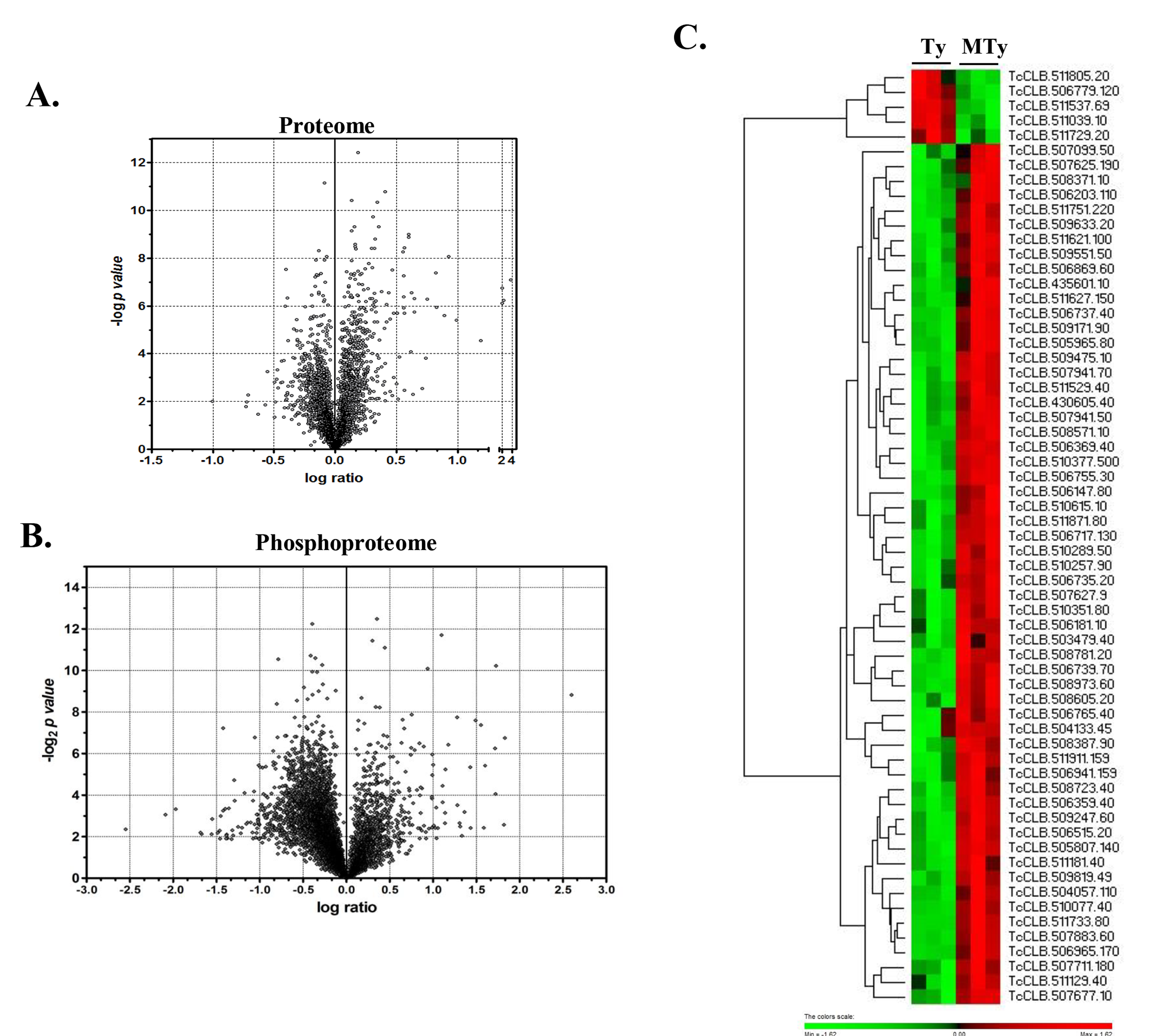

Supplement: S1 Fig — A. Volcano plot of total protein identified after LC-MS/MS. The Y-axis represents the -log 2 p-value (T-test) and X-axis represents the -log 2 Ratio of Protein intensity MTy/Ty. B. Volcano plot of total phosphopeptides identified after phospho-enrichment followed by LC-MS/MS. Y and X-axis are the same as represented in A. C. Heat map of the 64 proteins with significant differences between MTy and Ty samples. The Tritryp ID for each protein identified is indicated on the right. (TIF) [file pntd.0007103.s001.tif]

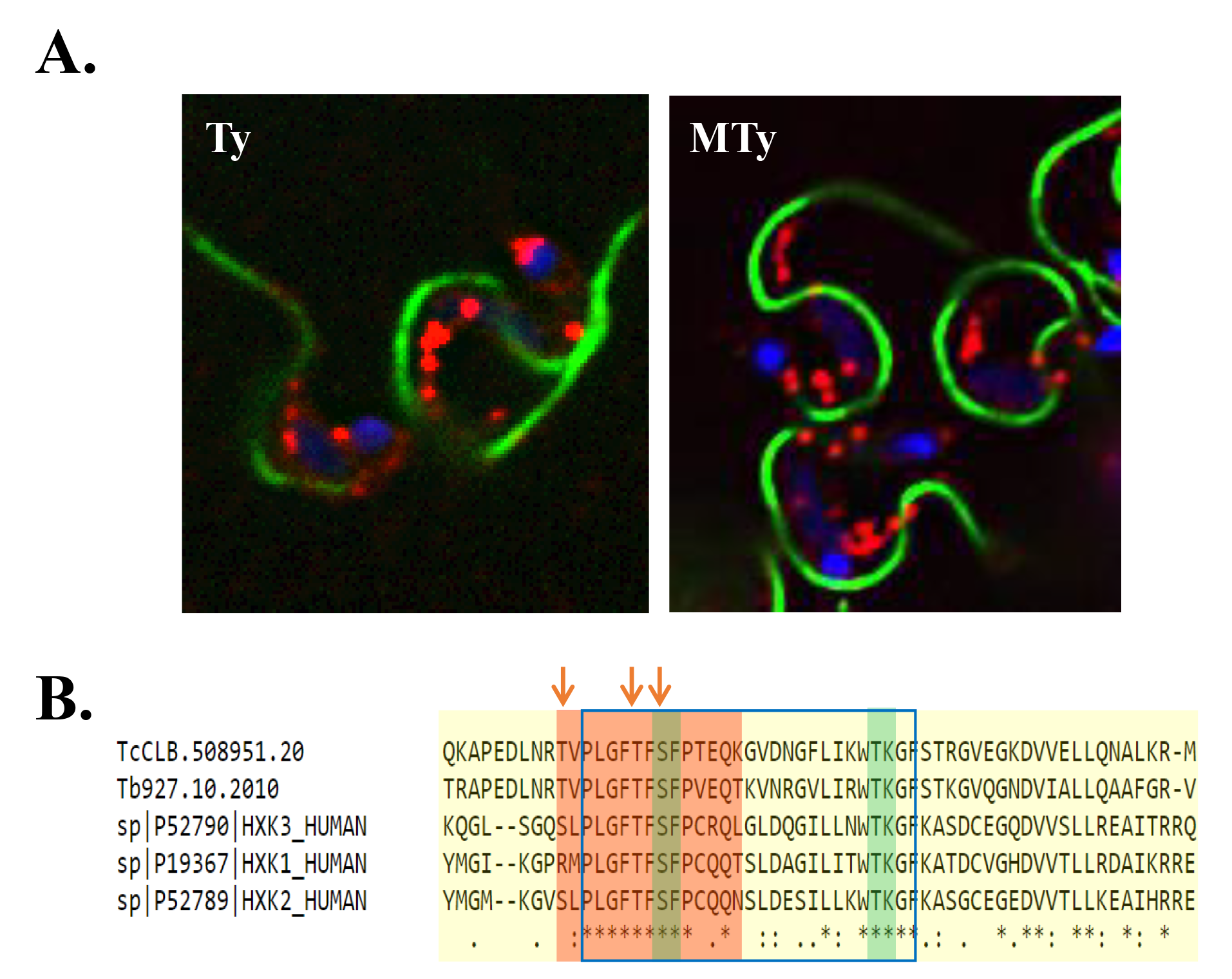

Supplement: S2 Fig — (A) Sub-cellular localization of Hexokinase (red), PFR (green) and nuclei (blue) in trypomastigotes. (B) Sequence aligment between isoforms of human-HKs (P52790; P19367; P52789) and Tc-HK (Q4D3P5/TcCLB.508951.20). The arrows indicate the phospho-residues and orange box indicates the phosphopeptide identified after phosphoproteome analysis. (TIF) [file pntd.0007103.s002.tif]

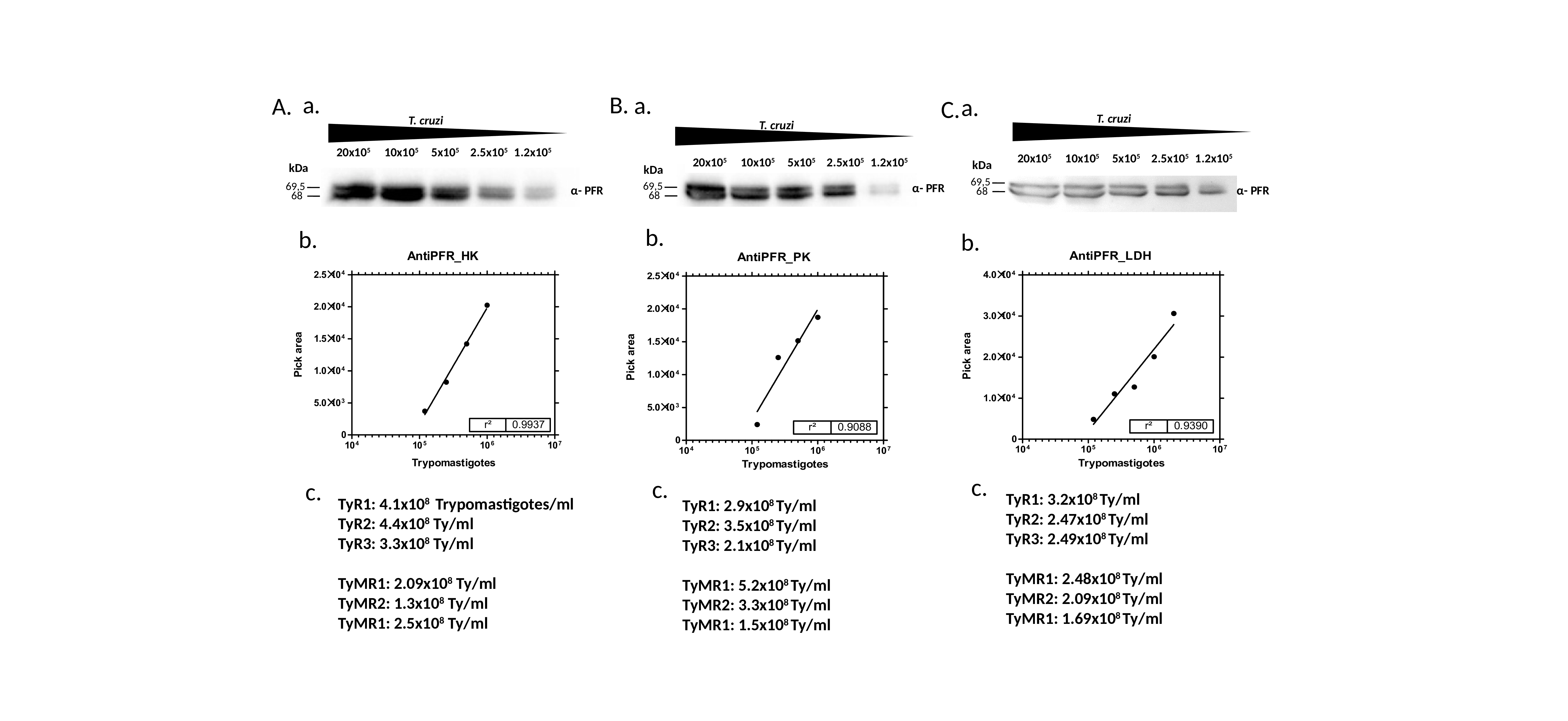

Supplement: S3 Fig — Correlation between the number of trypomastigotes and PFR loading for Ty and MTy extracts used for HK (A), PK (B) and LDH (C) enzymatic quantification assays. (a) Immunoblotting of 20 x105 to 1.2 x 10^5^ trypomastigotes extracts with antibody anti-Paraflagellar rod proteins (PFR). (b) Curve of linear correlation between curve area of the immunoblotting bands (a) and trypomastigote numbers. (c) Estimative of parasite number for each extract employed for enzymatic quantification assay shown in Fig 4 and S4 Fig. (TIFF) [file pntd.0007103.s003.tiff]

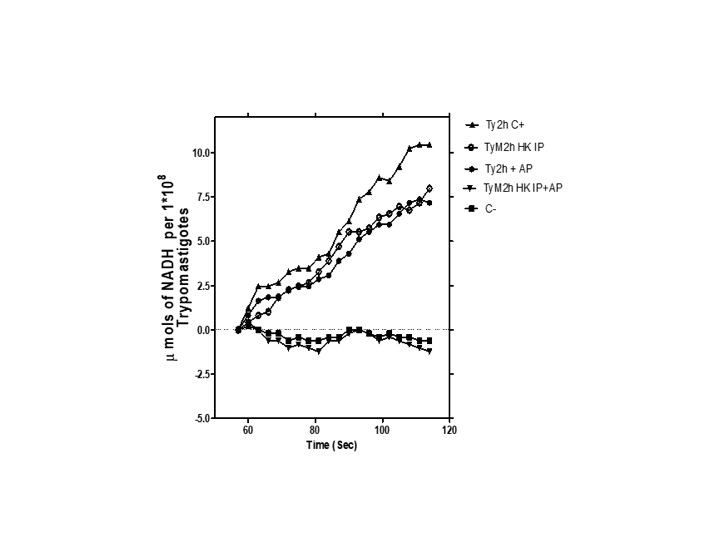

Supplement: S4 Fig — Extracts from parasites previously incubated with ECM for 2h (TyM2h) or with medium (Ty2hC, control) were immunoprecipitated with anti-HK antibodies (TyMHK IP and Ty2hC+), treated (+AP) or not with AP, followed by the measurement of HK activity. C- Ty extract. The number of parasites was based on the calibration curve presented in S Fig 3. (TIFF) [file pntd.0007103.s004.tiff]
